# miR-19a-3p and miR-19b-3p repress Nurr1 and Nur77 to promote microglial inflammation after spinal cord injury

**DOI:** 10.3389/fncel.2026.1783899

**Published:** 2026-03-23

**Authors:** Faezeh Sahebdel, Aliabbas Zia, Hector Ramiro Quintá, Andres Stucky, Leslie R. Morse, Julie K. Olson, Ricardo A. Battaglino

**Affiliations:** 1 Department of Physical Medicine and Rehabilitation, Miller School of Medicine, University of Miami, Miami, FL, United States; 2 Centre Hospitalier Universitaire Sainte-Justine, Montreal, QC, Canada; 3 Department of Pharmacology, Université de Montréal, Montreal, QC, Canada; 4 National Scientific and Technical Research Council (CONICET), Buenos Aires, Argentina; 5 Laboratorio de Medicina Experimental, “Dr. Toblli” – Hospital Aleman, Buenos Aires, Argentina; 6 Department of Diagnostics and Biological Sciences, School of Dentistry, University of Minnesota, Minneapolis, MN, United States; 7 Department of Orthopaedics, Miller School of Medicine, University of Miami, Miami, FL, United States

**Keywords:** miR-19a-3p, miR-19b-3p, neuroinflammation, Nur77, Nurr1, spinal cord injury, neuropathic pain

## Abstract

**Background:**

Spinal cord injury (SCI)-induced neuropathic pain affects up to 60% of individuals with SCI and is closely linked to microglia-driven neuroinflammation. Neuroinflammatory processes after SCI are major contributors to the development and persistence of chronic pain. MicroRNAs (miRNAs) have emerged as regulators of neuroinflammation. There are higher levels of circulating miR-19a and miR-19b in persons living with SCI with neuropathic pain compared to those with no pain. These miRNAs are associated with altered the neuroprotective genes Nurr1 and Nur77.

**Methods:**

Primary microglia cultures and a rat spinal cord injury model were used to investigate the regulatory effects of miR-19a and miR-19b on Nurr1 and Nur77 expression.

**Results:**

Our study shows that miR-19a and miR-19b and their binding sites in Nurr1’s 3′ UTR are highly conserved across vertebrates, suggesting functional importance. Through *in vitro* microglia cultures and *in vivo* rat SCI models, we demonstrate that these miRNAs negatively regulate Nurr1, Nur77, and inflammatory gene expression. Protein–protein interaction network analysis highlights transcription factors such as MYC, RUNX1, and STAT3 as central to this regulatory network.

**Conclusion:**

These findings support a model in which miR-19a and miR-19b contribute to microglia-driven neuroinflammation after SCI and highlight their potential as therapeutic targets to reduce neuropathic pain.

## 1 Introduction

Spinal cord injury (SCI) is a severe neurological injury with variable clinical outcomes depending on the location and severity of the lesion (Wilson et al., 2012). Damage to the spinal cord can cause varying degrees of motor and/or sensory impairments as well as paralysis (Hagen and Rekand, 2015). Pain is frequent among people living with a SCI and it is thought to be caused by damaged nerves affected by the SCI which can still send retrograde signals to the brain (Hagen and Rekand, 2015). Neuroinflammation, defined as an inflammatory reaction inside the neurological system, is a frequent complication of SCI. Neuroinflammation is a type of localized inflammation that affects the peripheral nervous system (PNS) and central nervous systems (CNS). The activation of glial cells in the dorsal root ganglia, spinal cord, and brain leads to the generation of pro-inflammatory cytokines and chemokines in the PNS and CNS, which induces peripheral and central sensitization (Matsuda et al., 2019). Understanding the processes of neuroinflammation and its relationship to pain in SCI is critical for developing effective treatment options to relieve pain and enhance the quality of life for people living with SCI.

Glial cells, which include oligodendrocytes, astrocytes, and microglia, have been linked to neuroinflammation and the development of neuropathic pain. Microglia, the CNS’s resident immune cells, initiate extensive inflammatory cascades in response to damage signals in combination with autocrine and paracrine signaling from astrocytes and neurons (Hanisch, 2002). These activated microglia and astrocytes contribute to the development of neuropathic pain. MicroRNAs (miRNAs) are short non-coding RNAs that control the flow of genetic information by influencing the translation or stability of mRNAs (Zhao and Srivastava, 2007). MiRNA dysregulation can lead to microglial hyperactivation, chronic neuroinflammation, and aberrant macrophage polarization in the brain. miRNAs act as modulators of neuroinflammation, as well as biomarkers, and have potential as targets for therapies aimed at treating CNS disorders (Guo et al., 2019). miRNAs can also alter inflammatory processes including neuroinflammation by influencing pro-inflammatory cytokine production. Previously, we discovered that SCI patients who experience neuropathic pain had higher levels of circulating miR-19a and miR-19b (Ye et al., 2021). In this recent research, we found 71 differentially expressed miRNAs in persons with chronic SCI and neuropathic pain compared to those without pain. Notably, two related miRNAs, hsa-miR-19a-3p and hsa-miR-19b-3p, were considerably upregulated in patients with SCI-associated neuropathic pain and had strong discriminatory capacity between pain and non-pain groups (Ye et al., 2021).

miRNAs are critical regulators of gene expression that function through conserved mechanisms across species (Bartel, 2004). Several human microRNAs are highly conserved across species, and many of these conserved miRNAs are clustered together in the genome, which suggests shared regulatory functions and evolutionary importance (Bentwich et al., 2005). Recent studies have highlighted the pivotal role of microRNAs in regulating key transcriptional pathways involved in cellular stress and neurodegeneration; for example, miRNA-mediated modulation of SIRT1 has been implicated in aging and age-associated diseases (Zia et al., 2021). miRNAs have also emerged as key regulators of aging and longevity by modulating cellular pathways and gene expression profiles across various tissues, and growing evidence highlights their involvement not only in age-related processes but also in cellular responses to neurological injury (Zia et al., 2022). The 3′- untranslated region (3’-UTR) of Nurr1 mRNA contains conserved seed sequences targeted by multiple miRNAs, including miR-204, miR-93, and miR-302d, which have been shown to regulate Nurr1 expression specifically in mesencephalic dopamine neurons (Pereira et al., 2017). MicroRNAs (miRNAs) show highly tissue-specific expression patterns across human organs, with certain miRNAs such as miR-122 enriched in the liver and miR-124 in the brain. This spatial regulation suggests a fundamental role of miRNAs in controlling tissue-specific gene expression and cellular identity (Ludwig et al., 2016).

Transcription factors Nurr1 and Nur77 may play a significant role in modulating neuroinflammation following SCI. For instance, in one study Nurr1 expression was found to be important in controlling inflammation in the brain (Lallier et al., 2016). Recent transcriptomic profiling of primary microglia has shown that miR-19a-3p and miR-19b-3p distinctly regulate inflammatory signaling pathways and transcription factors involved in microglial activation following spinal cord injury (Sahebdel et al., 2024). Another study revealed that in LPS-activated microglia, the expression of Nur77 changed the expression of several proteins implicated in microglial inflammation (Chen et al., 2017). The aim of this study is to investigate the potential role of miR-19a and miR-19b in the regulation of Nurr1 and Nur77 expression after SCI. We hypothesized that following SCI, increased levels of miR-19a and miR-19b result in decreased expression of Nurr1 and Nur77 ultimately resulting in microglia activation and neuroinflammation. We found that Nurr1 and Nur77 were expressed in microglia and that expression of Nurr1 and Nur77 was downregulated by miR-19a and miR-19b mimics. We also found that expression of Nurr1 and Nur77 was increased in the brains of SCI rats and decreased in SCI rats treated with Netrin-1, a protein that promotes axonal regeneration and exhibits anti-neuroinflammatory activity as was previously described (Quintá, 2021).

Overall, this study investigates at investigating the potential role of miR-19a and miR-19b in Nurr1 and Nur77 expression after spinal cord injury. We hypothesized that miR-19a and miR-19b reduce the expression of Nurr1 and Nur77 and increase the expression of proinflammatory cytokines and chemokines following spinal cord injury. By investigating the complex link between SCI, neuropathic pain, neuroinflammation, and the regulatory function of miRNAs in microglia activation, this research attempts to provide new insights into potential treatment options for people living with SCI.

## 2 Materials and methods

### 2.1 Microglia cultures

Primary microglia cells were isolated from SJL/J mice as previously described (Olson and Miller, 2004). Briefly, newborn mouse brains were dissected, minced, and digested to yield a cell suspension. Cells were grown as a mixed glia culture for 14 days. The cultures were then shaken for 24 h to separate the microglia. Microglia were grown in poly-D-lysine-coated 24 well plates with DMEM high-glucose media (Gibco, 11,965,092), 20% FBS, and 3 ng/mL rGM-CSF (R&D Systems). The M4T.4 microglia cell line was generated from primary microglia as previously stated (Olson et al., 2003). M4T.4 cells were seeded in T25 flasks covered with poly-D-lysine.

### 2.2 Microglia activation and transfection

We employed two models of microglial activation: IFN-*γ* stimulation and LPS stimulation. Microglia cells were stimulated with LPS (Sigma-Aldrich, 437,620) or interferon-gamma (rIFN-γ, I4777) (Sigma-Aldrich) for 24 h at 37 °C in the presence or absence of (1) miR-19a mimic (Qiagen, YM00472044), (2) miR-19a inhibitor (Qiagen, YI04101300), (3) miR-19b mimic (Qiagen, YM00470545), (4) miR-19b inhibitor (Qiagen, YI04101488), and (5) miR-19 mimic control (Qiagen, YM00479903). Control cultures were transfected with the same miRNAs, but they were not stimulated with LPS or rIFN-*γ*. Lipofectamine RNAiMAX (Invitrogen, #13778–030) was used to transfect miRNAs according to the manufacturer’s instructions.

### 2.3 SiRNA transfection and gene silencing *in vitro*

Silencing of Nurr1 and Nur77 expression was monitored *in vitro* in M4T.4 cells. Cells were plated in T25 flasks for 24 h before transfection. Nurr1 siRNA (Horizon, ON-TARGETplus siRNA, L-048281-01-0005) or Nur77 siRNA (Horizon, ON-TARGETplus siRNA, L-040970-01-0005) was added to the cells. Cells were then stimulated with LPS or IFN-γ (Sigma-Aldrich). SiRNAs were transfected using DharmaFECT 4 Transfection Reagent (Horizon, T-2004-02) according to the manufacturer’s instructions. After incubation for 24 h at 37 °C, the cells were lysed, and total RNA was isolated.

### 2.4 RNA isolation and real time PCR analysis

After incubating the microglia cells for 24 h, we used the SV Total RNA Isolation kit (Promega, #Z3100) to lyse and isolate total RNA. The Advantage for RT-PCR kit (Clontech Laboratories, Takara Bio Company, #639506) was used to generate cDNA from 2 μg of microglia total RNA using oligo 12–18 primers in a final volume of 100 μL. For real time PCR, the LightCycler-Faststart DNA Master SYBR Green I kit (Roche Molecular Biochemicals) was utilized. PCR was performed using 2 mM MgCl2, 0.5 μM primers, 1x FastStart DNA SYBR Green I reagent, and 2 μL of diluted cDNA. Primers were used for Nurr1, Nur77, TNF-*α*, IL-6, and IL-12. Real time PCR was performed using the Rotorgene 2000 using the following program: 40 cycles of 15 s in 95 °C, 2 s in 60 °C, and 15 s in 72 °C, followed by a cycle from 75 °C to 95 °C and 5 min in 72 °C for the final extension. We used *β*-actin as a housekeeping gene. The 2^(−ΔΔCt) comparative cycle threshold approach was utilized to determine the fold change in expression.

### 2.5 Statistical analysis

For each experiment, three biological replicates (*n* = 3) were performed. The real time PCR was done in triplicate, and the results were determined using the mean +/− SD. A one-way ANOVA analysis and the Bonferroni comparison test were used to determine if there were significant differences between stimulated and unstimulated microglia groups. A *p*-value < 0.05 was considered statistically significant.

### 2.6 Animals

Healthy adult male rats (8–10 weeks old) weighing 200–250 g are provided by Laboratorio de Medicina Experimental, “Dr. Jorge Toblli”: Hospital Alemán, Buenos Aires, Argentina. The animal experimentation was approved by the Ethics Committee of “Sociedad Argentina de Investigación Clínica – SAIC” (Register code: 03–22) and was conducted according to the United States National Institutes of Health Guide for the Care and Use of Laboratory Animals. Dr. Quintá performed the experiments at the Experimental medicine lab, in the hospital alemán, Argentina. The animals received a lateral contusion-compression injury in the spinal cord at the Th11-Th12 lamina level by extradural application of an aneurysm clip with 70 g closing force during 120 s, using a modification of technique that was previously described by Scarsibrick’s Lab (Kim et al., 2021). One month after injury (chronic injury stage), Netrin-1 was injected into the spine of injured rats, as was previously described (Schmidt et al., 2025). Control rats were sham-operated (sham). Duration of the whole experiment corresponds to 105 days.

### 2.7 Immunofluorescent staining

Unstained sections (4 μm) were de-paraffinized and rehydrated using standard methods. For antigen retrieval, slides were placed in 6.0 pH buffer (Reveal Decloaking reagent, Biocare Medical, Concord, CA) in a steamer for 30 min at 95–98 °C, followed by a 20 min cool down period. All slides were then placed into a TBST rinse for 5 min. Endogenous peroxidase activity was quenched by slide immersion in 3% hydrogen peroxide/TBST solution followed by a 10-min tap water rinse and back into TBST. A serum-free blocking solution (Rodent Block R, Biocare Medical, Concord, CA) was placed on sections for 1 h RT. Blocking solution was removed and slides were incubated in primary antibody diluted in 10% blocking solution/90% TBST. Day 1 Nurr1 (abcam, Boston, MA, Clone N1404) 1:100 and anti-Iba-1 (WAKO, Richmond, VA, Clone E404W,1:2 K) were cocktailed, applied to the slides and incubated overnight at 4 degrees C. The following morning, they were placed into TBST, 3 rinses for 10 min each. The immunofluorescent secondary’s antibodies were cocktailed and applied. (Nurr1) anti-ms AF488 and (Iba-1) anti-Rb AF647 (Both 1:1 K). The secondary was incubated at room temperature for 2 h followed by TBST rinses, 3 × 10 min each. Following the rinse, Rodent Block R (Biocare, Concord, CA) was placed on the slides for 30 min at room temperature. The block was drained and Nur77 (abcam, Boston, MA) 1:400 was applied and slides were incubated overnight at 4 degrees. The following morning the slides were placed into a TBST rinse, 3 x for 10 min each. The anti-Rabbit AF594 (1,500) was then applied and incubated for 2 h at room temperature. This incubation was followed by TBST rinses, 3 × 10 min each. The slides then went into two rinses of distilled water and cover slipped with ProLong Diamond Antifade mountant with DAPI.

### 2.8 Sequence conservation and target prediction

To assess sequence conservation, we used seed-based target prediction tools, including TargetScan and PicTar, to identify conserved binding sites for miR-19a-3p and miR-19b-3p in the 3′ UTR of the Nurr1 (NR4A2) gene across multiple vertebrate species.

### 2.9 miRNA expression and pathway enrichment analysis

Expression data for miR-19a-3p and miR-19b-3p were obtained from publicly available databases such as miTED (miRNA Tissue Expression Database) and the miRNA Tissue Atlas Database. Co-expression analysis was performed using Spearman correlation. Functional enrichment of predicted target genes was carried out using miRPath v3.0 with both KEGG and Reactome pathway databases.

### 2.10 Protein–protein interaction network analysis

Predicted target genes of miR-19a and miR-19b were analyzed using the STRING v12.0 database to construct protein–protein interaction (PPI) networks. High-confidence interactions were included, and node degree was used to identify key transcription factors involved in the network.

## 3 Results

### 3.1 The evolutionary conserved sequences and targets of miR-19a-3p and miR-19b-3p across different organisms

**Figure 1:**
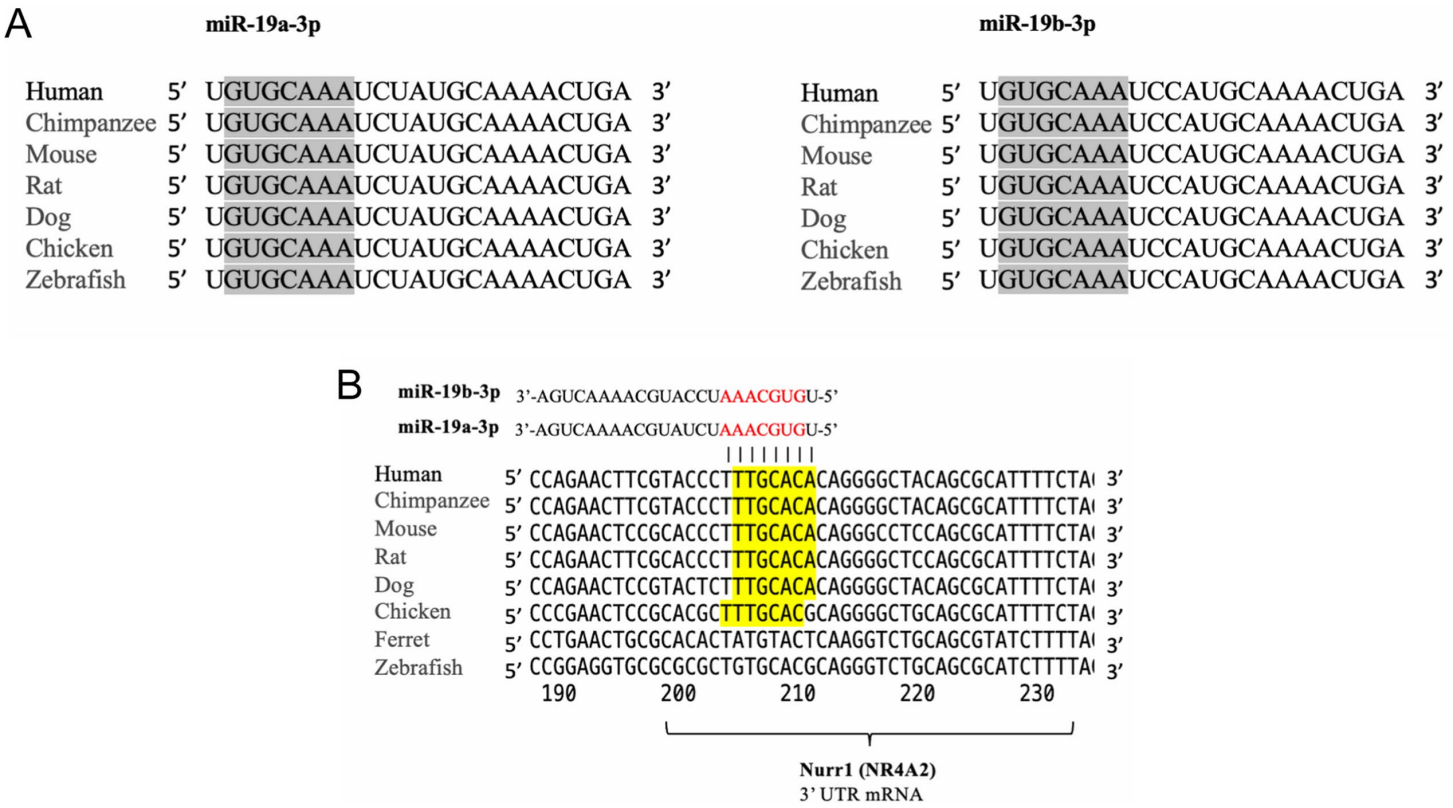
miR-19a-3p and miR-19b-3p are highly conserved in vertebrates. **(A)** Sequence alignment of mature miR-19a-3p and miR-19b-3p across multiple species, including humans, chimpanzees, mice, rats, dogs, chickens, and zebrafish. Both miRNAs exhibit high conservation among vertebrates. **(B)** Predicted binding sites of miR-19a-3p and miR-19b-3p within the 3′ UTR of Nurr1 (NR4A2) mRNA across various species. The seed match region is highlighted in yellow and shows strong sequence conservation in mammals, which suggests a conserved post-transcriptional regulatory role of miR-19a and miR-19b in modulating Nurr1 expression.

We conducted a comprehensive bioinformatics analysis to investigate the conservation of miR-19a-3p and miR-19b-3p sequences and their potential targets across different organisms. Using seed-based target prediction algorithms, specifically, PicTar and TargetScan databases, we identified conserved miRNA sequences and their corresponding mRNA targets to finally detect conserved 8mer, 7mer, or 6mer sites that match the seed region of miR-19a and miR-19b. This allowed us to determine the evolutionary conservation of miR-19a-3p and miR-19b-3p in various species. Our analysis revealed a high degree of conservation for miR-19a-3p and miR-19b-3p sequences among mammals and vertebrates (Figure 1A). By applying seed-based target prediction methods, we further identified conserved binding sites in the 3′ UTR of Nurr1 mRNA across multiple species. Notably, we found a highly conserved heptameric seed sequence at the 3′ UTR of Nurr1 mRNA in mammals and vertebrates that suggests a strong evolutionary pressure to maintain this regulatory interaction (Figure 1B).

### 3.2 Tissue-specific expression and functional pathway enrichment of miR-19a and miR-19b: insights into immune regulation and neural signaling

**Figure 2:**
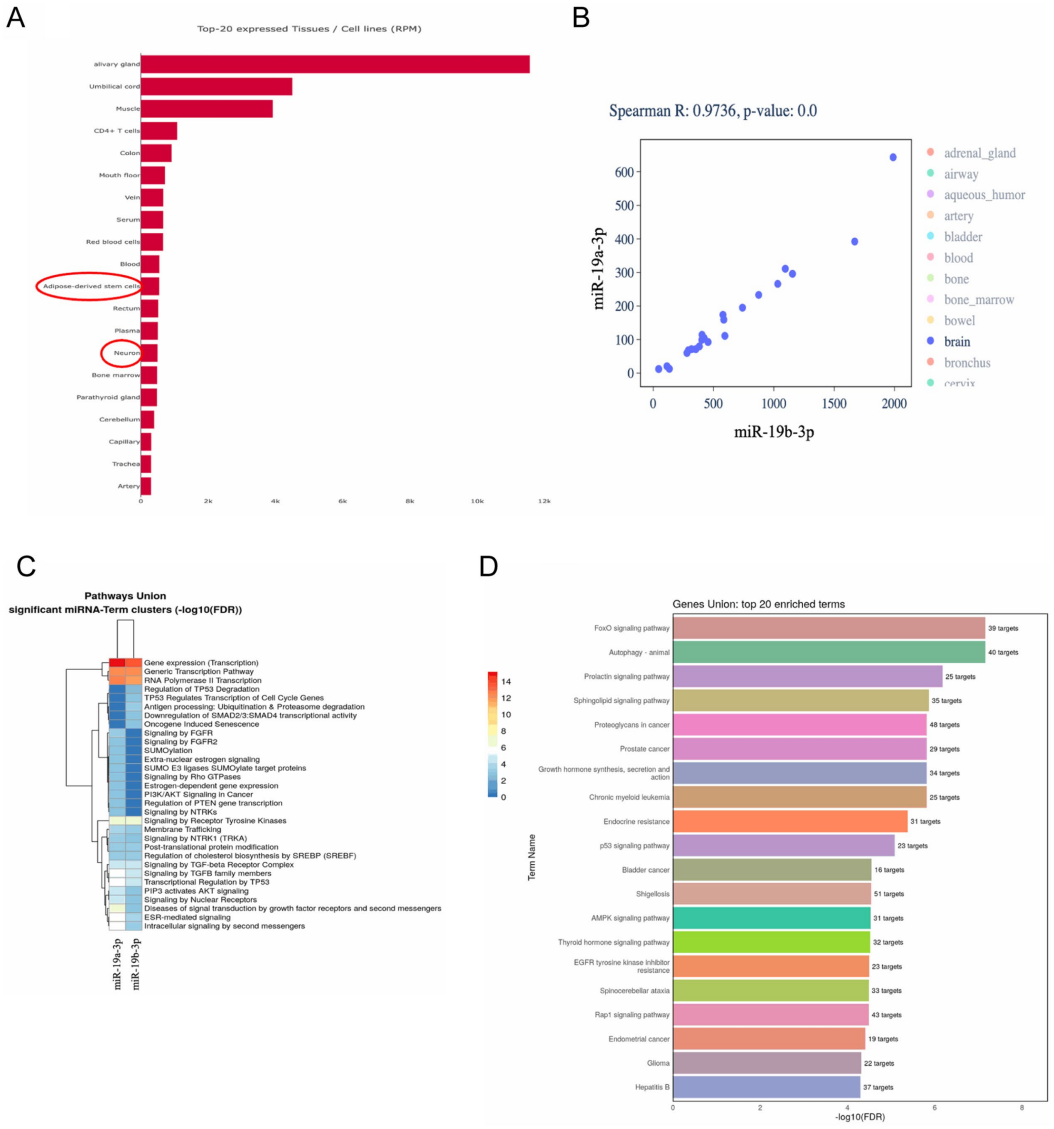
miR-19a-3p and miR-19b-3p are highly expressed in neural and immune tissues, co-expressed across tissues, and linked to transcriptional, inflammatory, and neurodegenerative pathways. **(A)** Bar chart illustrating the top 20 tissues and cell types with the highest expression levels of miR-19a and miR-19b, showing predominant enrichment in the salivary gland, umbilical cord, muscle, CD4 + T cells, and neuron-rich tissues. **(B)** Scatter plot showing co-expression levels of miR-19a-3p and miR-19b-3p across tissues. A strong positive correlation was observed (Spearman R = 0.9736, *p* = 0.001) with brain samples. **(C)** Reactome pathway analysis of predicted target genes for miR-19a-3p and miR-19b-3p using miRPath (FDR < 0.001) revealed significant enrichment in transcriptional regulation, signaling by receptor tyrosine kinases, and PI3K/AKT signaling. The color bar reflects the −log10^(*p*-value)^, with red representing higher values and blue representing lower values. **(D)** KEGG pathway analysis showing the top 20 enriched pathways targeted by miR-19a and miR-19b (FDR < 0.001).

We analyzed miR-19a and miR-19b expression profiles across a wide range of human tissues and cell types to gain insight into the biological relevance of these miRNAs. Both miRNAs showed notably high expression in salivary glands, umbilical cord, muscle, and immune-related tissues such as CD4 + T cells. Moreover, neuronal tissues and adipose-derived stem cells also express high levels of miR-19a and miR-19b (Figure 2A), which suggests a potential role in both immune responses and neuroregeneration. Co-expression analysis showed a strong correlation between miR-19a-3p and miR-19b-3p across multiple tissues (Spearman *R* = 0.9736, *p* = 0.001), especially in brain samples (Figure 2B).

We performed pathway enrichment analysis using the miRPath database to explore the functional impact of miR-19a and miR-19b. Reactome-based analysis revealed significant enrichment in pathways related to gene expression, transcriptional regulation, SUMOylation, and signaling cascades such as PI3K/AKT and FGFR (Figure 2C). Kyoto Encyclopedia of Genes and Genomes (KEGG) pathway analysis further identified strong associations with FoxO signaling, autophagy, glioma, endocrine resistance, and various inflammation and cancer-related pathways (Figure 2D). These results suggest that miR-19a-3p and miR-19b-3p may modulate critical signaling networks involved in transcription, immune regulation, and neuronal survival, supporting their relevance in spinal cord injury pathophysiology.

### 3.3 Treatment with miR-19a and miR-19b reduces expression of Nurr1 and Nur77

In order to determine whether miR-19a and miR-19b could directly affect Nurr1 and Nur77 expression, microglia cells were transfected with miR-19a mimic, miR-19b mimic, miR-19a inhibitor, miR-19b inhibitor, or negative control miR mimic. In a parallel set of experiments, microglia were stimulated with LPS (5 μg/mL) or IFN-*γ* (100 U/mL) and transfected with the same miRs. Twenty-four hours later, microglia were analyzed for the expression of Nurr1 and Nur77 by real-time PCR.

**Figure 3:**
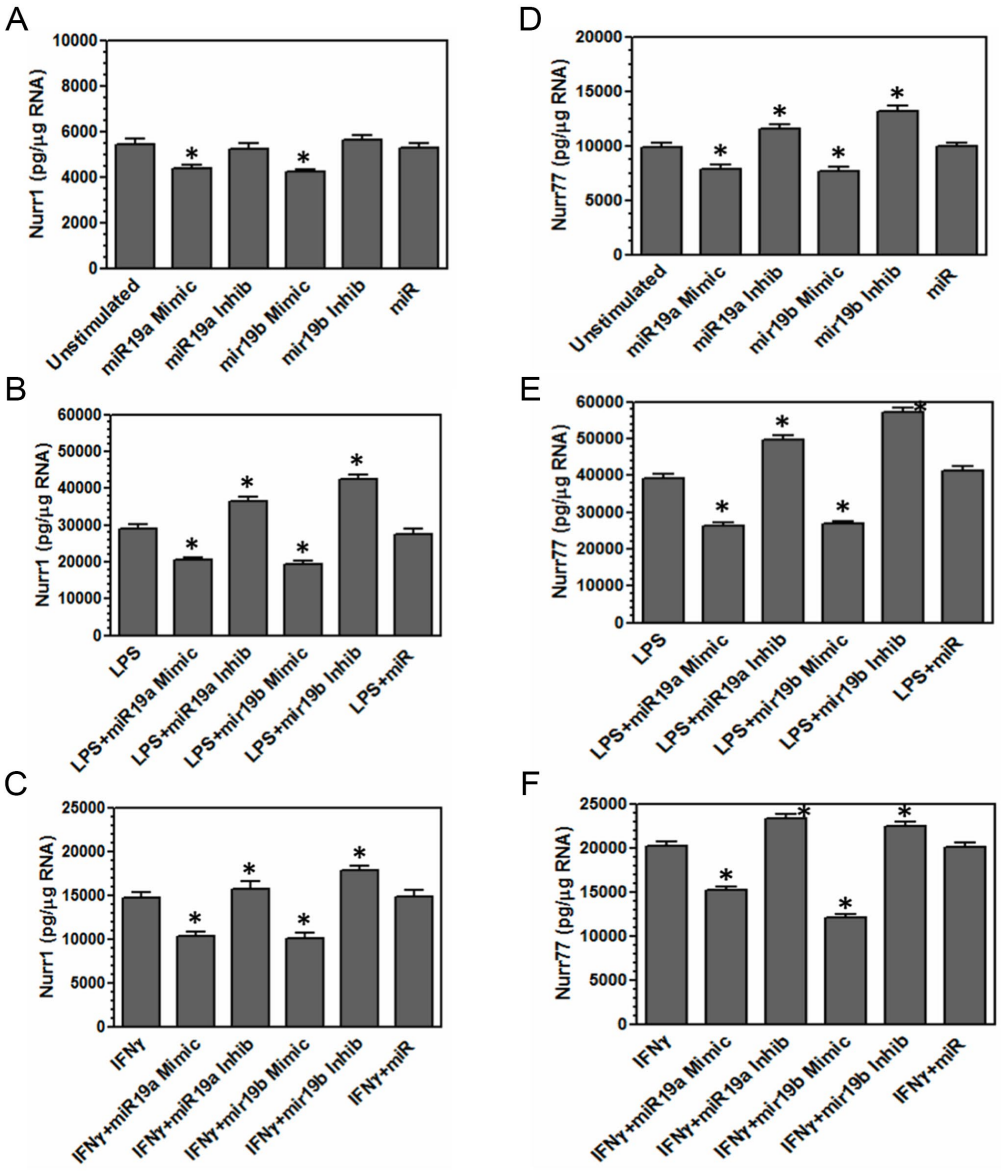
miR-19a and miR-19b decrease the expression of Nurr1 and Nur77 in microglia. Microglia were transfected with miR-19a mimic, miR-19b mimic, miR-19a inhibitor, miR-19b inhibitor or negative control miR mimic **(A,D)**. Moreover, microglia were stimulated with LPS (5 μg/mL) or IFN-*γ* (100 U/mL) and transfected with miR-19a mimic, miR-19b mimic, miR-19a inhibitor, miR-19b inhibitor, or negative control miR mimic **(B,C,E,F)**. After 24 h, microglia were lysed and RNA was isolated. RNA was converted to cDNA and used in real time PCR with primers for Nurr1 and Nur77. The concentration was based on standards for each set of primers, and groups were normalized based on the expression of β-actin. A significant difference (*) was determined by one-way ANOVA and Bonferroni’s multiple comparison tests (*p < 0.001) based on unstimulated microglia. These are representative graphs from one experiment of three independently repeated experiments.

Treatment with miR-19a and miR-19b mimics resulted in decreased expression of Nurr1 and Nur77 in unstimulated and LPS- and IFN-γ-stimulated cells compared to control untreated microglia (Figures 3A,D). Treatment with miR-19a and miR-19b inhibitors, on the other hand, resulted in increased expression of Nur77 in unstimulated microglia and both Nurr1 and Nur77 in microglia stimulated with LPS or IFN-γ compared to control untreated microglia (Figures 3B,C,E,F). These results suggest that both miR-19a and miR-19b can affect the expression of transcription factors Nurr1 and Nur77.

### 3.4 Expression of Nurr1 and Nur77 decreases in microglia following spinal cord injury

**Figure 4:**
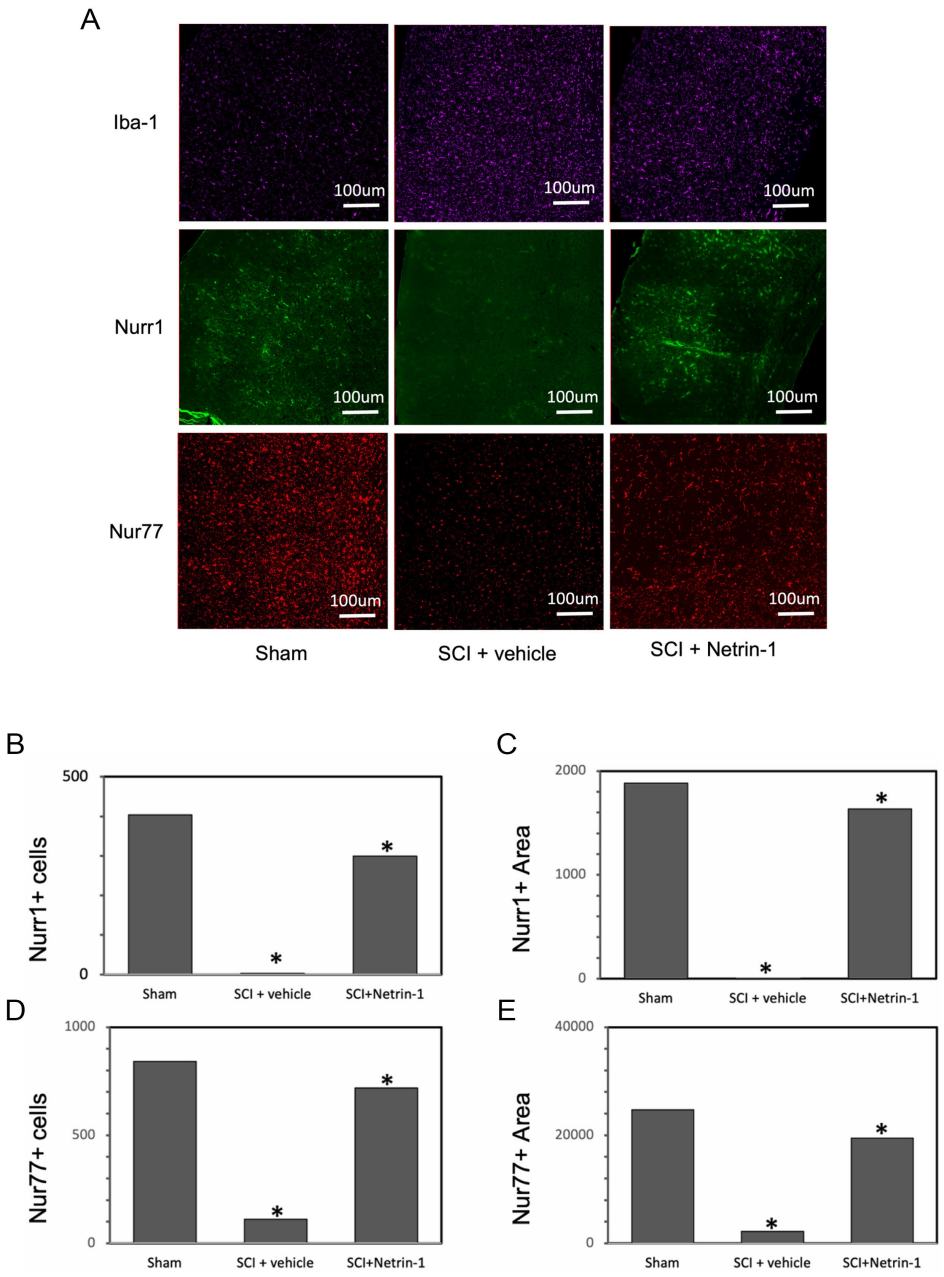
Expression of Nurr1 and Nur77 is decreased in the brains of SCI vehicle treated-rats. Brains from four animals per group (sham, SCI + vehicle, and SCI + Netrin-1) were analyzed by Immunofluorescence. Representative high-magnification images illustrate the expression of Iba-1, Nurr1, and Nur77 in rat brains **(A)**. Iba-1 staining was used as a marker for detecting both resting and activated microglia in the brain. Immunofluorescence showing differentially expressed Iba-1, Nurr1, and Nur77 in rats. Number of Iba-1 + cells were significantly increased in SCI vehicle treated-rats and SCI-rats treated with Netrin-1 compared to the sham while the expression of Nurr1 + and Nur77 + cells were significantly decreased in SCI vehicle treated-rats compared to sham and SCI-rat treated with Netrin-1 **(A)**. The bar charts are showing the number and area of the Nurr1 + **(B,C)** and Nur77 + **(D,E)** cells that are significantly lower in SCI vehicle treated-rats compared to the sham and SCI + Netrin-1. Immunofluorescence staining shows Iba-1 in purple, Nurr1 in green, and Nur77 in red.

**Figure 5:**
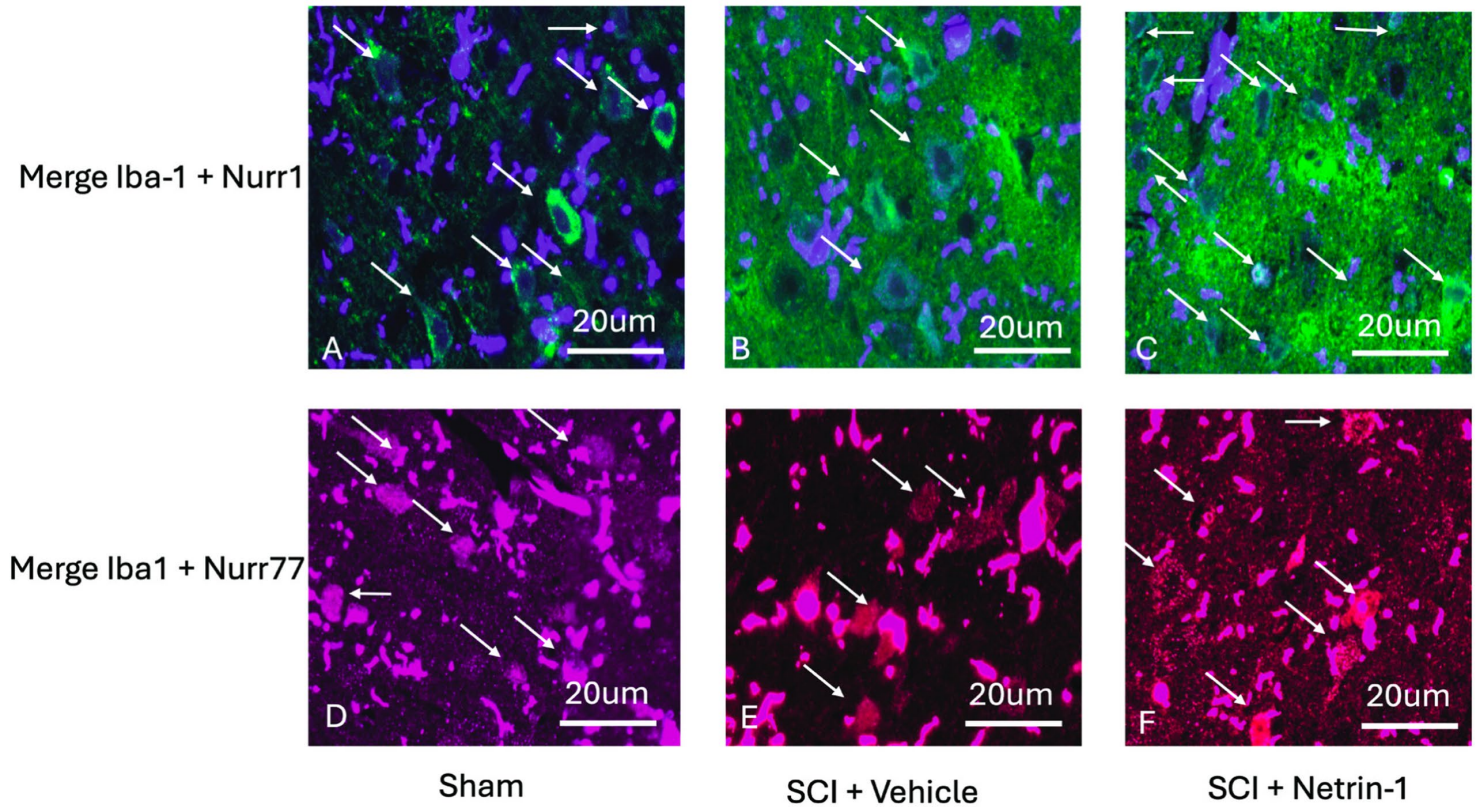
Nurr1 and Nur77 co-localize with the microglial marker Iba-1. Representative high-magnification immunofluorescence images showing co-localization of Nurr1 with Iba-1 **(A–C)** and Nur77 with Iba-1 **(D–F)** in the brains of Sham **(A, D)**, SCI + vehicle **(B, E)**, and SCI + Netrin-1–treated rats **(C, F)** (*n* = 4 animals per group). White arrows indicate regions of signal overlap between Nurr1 or Nur77 and Iba-1. Because Iba-1 is specifically expressed in microglia, this co-localization demonstrates that Nurr1 and Nur77 are expressed in microglial cells. Merged images are shown to illustrate spatial overlap between transcription factors and the microglial marker. Immunofluorescence staining shows Iba-1 in purple, Nurr1 in green, and Nur77 in red.

Our goal was to determine how the expression of Nurr1 and Nur77 changed in the microglia of the brains of spinal cord-injured rats. For that, we analyzed the brains of three experimental groups (Sham, SCI + vehicle, and SCI + Netrin-1), with four animals per group (*n* = 4 per group) by Immunofluorescence. Since Netrin-1 is an axon guidance protein that has the ability to suppress the inflammatory response in SCI (Quintá, 2021), this was used as a second control. Iba-1 staining was used as a marker for detecting both resting and activated microglia in the brain. The results showed that the expression of Iba-1 increased in the SCI vehicle-treated rats compared to sham and SCI rats treated with Netrin-1 (Figure 4A). Immunofluorescence staining showed Iba-1 in purple, Nurr1 in green, and Nur77 in red. Quantitative analysis revealed a significant reduction in both the number and total area of Nurr1^+^ (Figures 4B,C) and Nur77^+^ (Figures 4D,E) cells in the SCI + vehicle group compared to the sham and SCI + Netrin-1 groups. Moreover, the results indicated that Iba-1 and Nurr1 are expressed in the same cells in sham, SCI + vehicle, and SCI + Netrin-1 groups (Figures 5A–C). Furthermore, Iba-1 and Nur77 are expressed in almost the same cells in these three groups, and they are co-localized (Figures 5D–F). Given that Iba-1 is a well-established microglial marker, the observed co-localization of Iba-1 with Nurr1 or Nur77 indicates that these transcription factors are expressed within microglial cells.

### 3.5 Silencing of Nurr1 and Nur77 enhances pro-inflammatory cytokine expression in microglia

**Figure 6:**
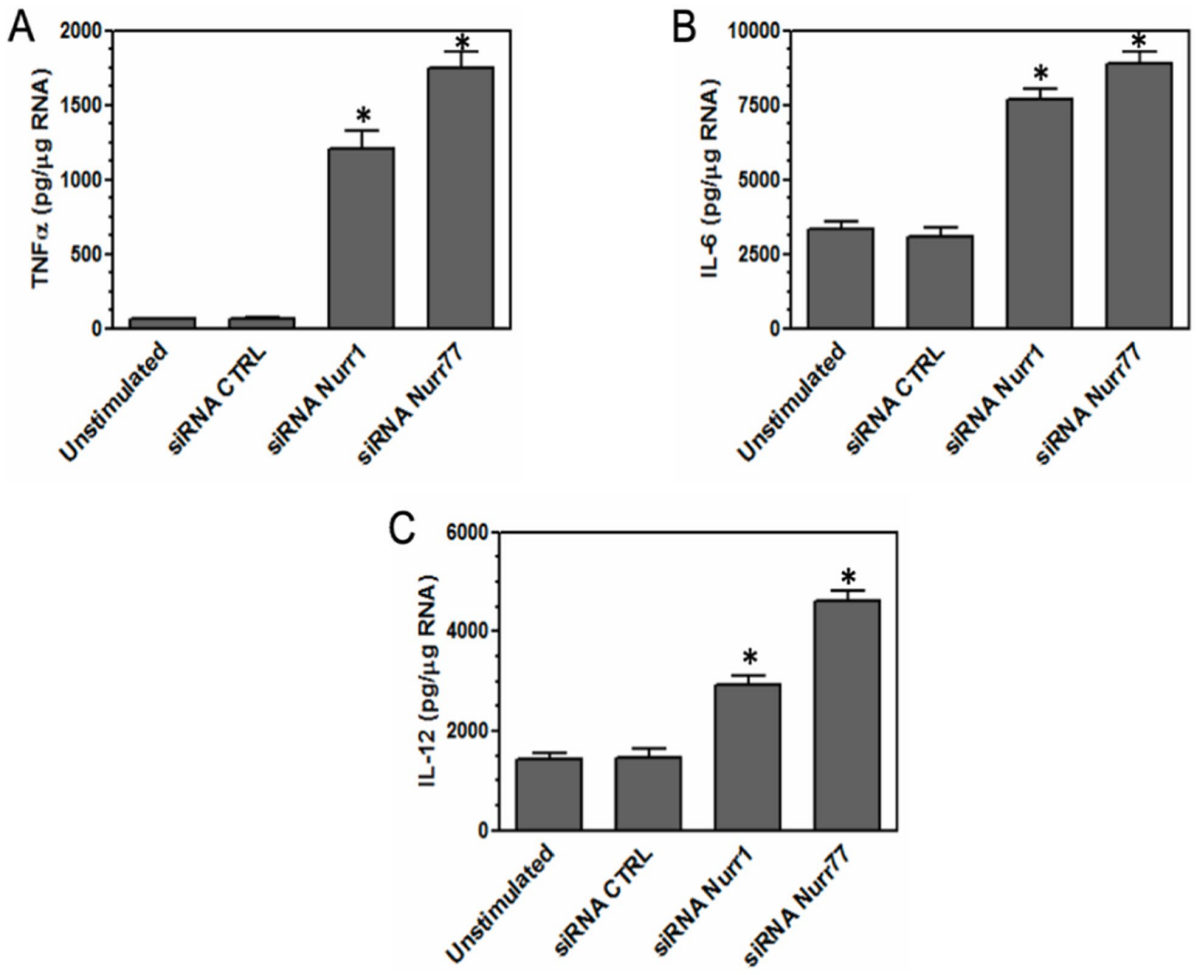
Silencing Nurr1 and Nur77 expression in microglia leads to an increased pro-inflammatory response. Microglia were transfected with control, Nurr1, and Nur77 siRNAs. After 24 h, microglia were lysed, and RNA was isolated. RNA was converted to cDNA and used for real time PCR with primers for TNF-α, IL-6, and IL-12. The concentration was based on standards for each set of primers, and groups were normalized based on the expression of β-actin. Silencing Nurr1 and Nur77 expression in microglia caused an increase in pro-inflammatory cytokines such as TNF-α **(A)**, IL-6 **(B)**, and IL-12 **(C)**. A significant difference (*) was determined by one-way ANOVA and Bonferroni’s multiple comparison tests (*p < 0.001) based on unstimulated microglia. These are representative graphs from one experiment of three independently repeated experiments.

To investigate the role of Nurr1 and Nur77 in regulating microglial inflammation, we silenced Nurr1 and Nur77 in microglia. Microglia were transfected with siRNAs for control, Nurr1, and Nur77. After 24 h, the expression levels of pro-inflammatory cytokines TNF-*α*, IL-6, and IL-12 were measured by real-time PCR. The concentration was based on standards for each set of primers, and groups were normalized based on *β*-actin expression. The results showed that silencing Nurr1 and Nur77 in microglia significantly increased cytokine expression, including TNF-α, IL-6, and IL-12, indicating a regulatory anti-inflammatory role for these transcription factors in microglia (Figures 6A–C).

### 3.6 Protein–protein interaction (PPI) network analysis of transcription factors targeted by miR-19a and miR-19b

**Figure 7:**
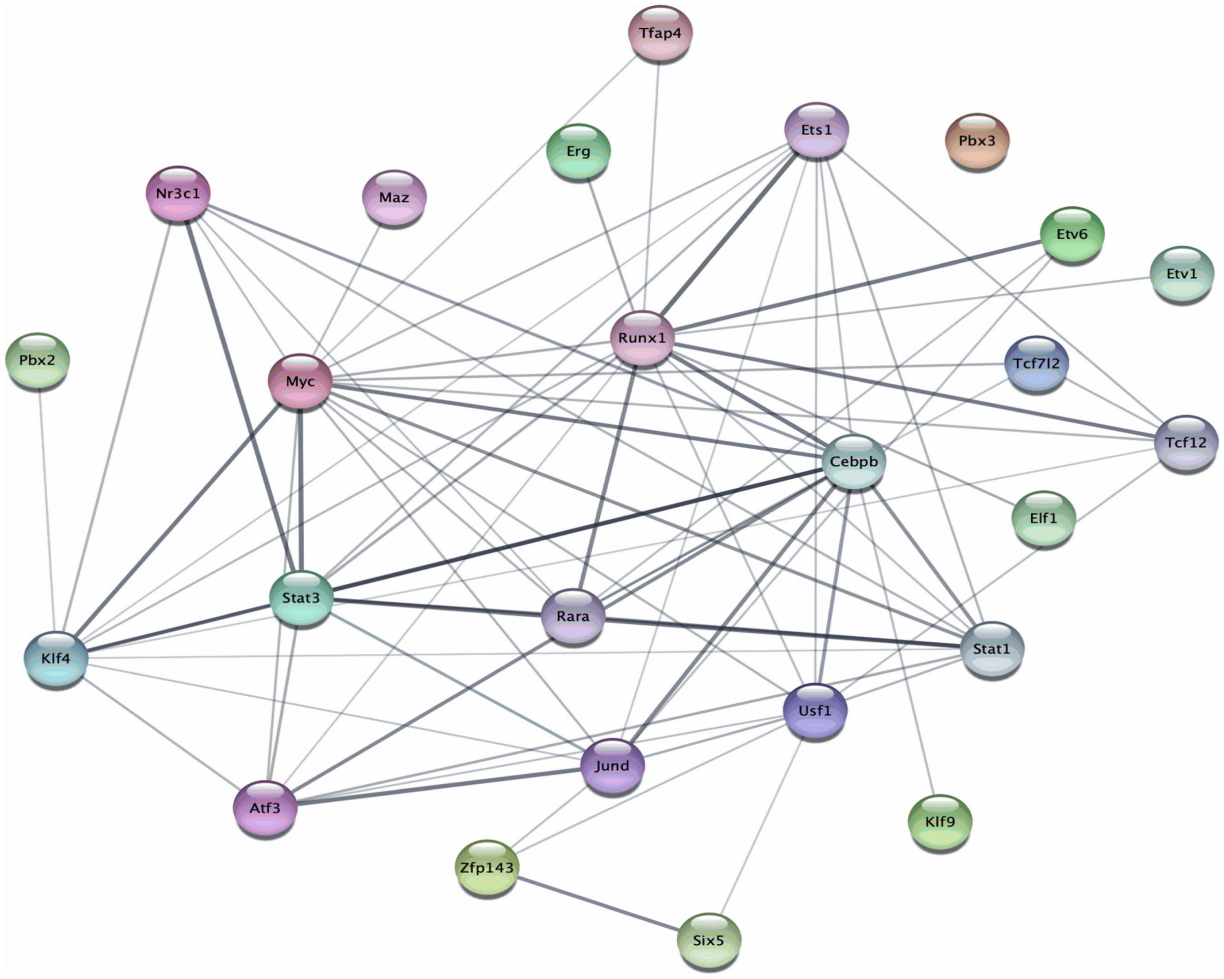
miR-19a and miR-19b are associated with a transcription factor interaction network revealed by Protein–Protein Interaction (PPI) network analysis. The STRING Protein–Protein Interaction Network (Version 12.0) tool was used to create networks of interactions between key transcription factors (TFs) related to genes that are regulated by miR-19a and miR-19b. The line thickness indicates the strength of the interaction.

We used the STRING database and created a protein–protein interaction (PPI) network that involves important transcription factors (TFs) that are regulated by miR-19a or miR-19b. The PPI network analysis of genes predicted to be regulated by miR-19a and miR-19b identified several highly connected transcription factors. Central nodes in the network included MYC, STAT3, RUNX1, CEBPB, and RARA, which exhibited strong interconnections with other regulatory proteins (Figure 7). These core hubs suggest coordinated regulation among genes involved in transcriptional control, immune responses, and cellular differentiation. The table shows specific transcription factors with their node degrees regulated by miR-19a and miR-19b according to the protein–protein interaction networks (Table 1).

**Table 1:** Top 25 transcription factors (TFs) regulated by miR-19a and miR-19b.

Top 25 TFs	Node degree
MYC	15
CEBPB	12
RUNX1	11
STAT3	10
KLF4	10
STAT1	10
USF1	10
ETS1	9
JUND	9
RARA	7
ATF3	7
NR3C1	6
TCF12	5
TFAP4	4
ETV6	3
ZFP143	3
TCF7L2	3
SIX5	2
PBX2	1
KLF9	1
ELF1	1
ETV1	1
MAZ	1
ERG	1
PBX3	0

The table shows an analysis of protein–protein interaction networks of genes regulated by miR-19a and miR-19b, identifying key transcription factors along with their corresponding node degrees.

MYC (MYC proto-oncogene, bHLH transcription factor), CEBPB (CCAAT/enhancer binding protein beta), RUNX1 (RUNX family transcription factor 1), STAT3 (signal transducer and activator of transcription 3), KLF4 (Krüppel-like factor 4), STAT1 (signal transducer and activator of transcription 1), USF1 (upstream transcription factor 1), ETS1 (ETS proto-oncogene 1, transcription factor), JUND (JunD proto-oncogene, AP-1 transcription factor subunit), RARA (retinoic acid receptor alpha), ATF3 (activating transcription factor 3), NR3C1 (nuclear receptor subfamily 3 group C member 1), TCF12 (transcription factor 12), TFAP4 (transcription factor AP-4), ETV6 (ETS variant transcription factor 6), ZFP143 (zinc finger protein 143), TCF7L2 (transcription factor 7 like 2), SIX5 (SIX homeobox 5), PBX2 (PBX homeobox 2), KLF9 (Krüppel-like factor 9), ELF1 (E74 like ETS transcription factor 1), ETV1 (ETS variant transcription factor 1), MAZ (MYC-associated zinc finger protein), ERG (ETS-related gene, ETS transcription factor), PBX3 (PBX homeobox 3).

## 4 Discussion

Neuroinflammation caused by microglia cells is a central contributor to neuropathic pain following SCI. Although several transcriptional regulators have been implicated in shaping microglial inflammatory phenotypes, the post-transcriptional mechanisms controlling these pathways remain incompletely understood. In this study, we investigated the role of miR-19a and miR-19b in regulating neuroinflammation through their impact on the transcription factors Nurr1 (NR4A2) and Nur77 (NR4A1), both of which are known to contribute to anti-inflammatory and neuroprotective signaling in microglia. We hypothesized that miR-19a and miR-19b modulate Nurr1 and Nur77 expression and thereby influence inflammatory responses in the central nervous system. Our findings showed that miR-19a and miR-19b suppress Nurr1 and Nur77 expression in microglia, leading to increased pro-inflammatory signaling after spinal cord injury.

Our results showed that microRNAs have a high degree of evolutionary conserved sequences and their expression varies significantly across different tissues, which reflects their distinct regulatory roles in tissue-specific gene expression and function. We analyzed the top 20 tissues and cell lines with the highest expression levels of miR-19a and miR-19b. Our analysis revealed that both miR-19a and miR-19b are highly expressed in immune-related tissues, including CD4 + T cells, as well as neural tissues such as the brain and adipose-derived stem cells, which suggests a shared regulatory role across immune and neural systems. Target prediction and enrichment analysis suggested miR-19a and miR-19b significantly impact transcriptional regulation and receptor tyrosine kinase signaling, consistent with their role in cellular proliferation.

MiRNAs are associated with several signaling pathways related to inflammation and cellular regulation. The KEGG analysis of the top 20 enriched pathways targeted by miR-19a and miR-19b showed these miRs were involved in the important neuroinflammatory pathways such as FoxO signaling pathway, autophagy, PI3K/AKT signaling pathway, p53 signaling pathway, AMPK signaling pathway. One study showed that FoxO transcription factors are involved in oxidative stress responses, immune regulation, and apoptosis (Webb and Brunet, 2014), all of which are key in neuroinflammation and are known to interact with NR4A family members. Autophagy influences neurodegenerative disease progression and inflammation (Alirezaei et al., 2011) and Nurr1 is known to regulate autophagy-related genes (Zarei et al., 2021). Since Nurr1 is a key regulator of autophagy and anti-inflammatory responses, and miR-19a and miR-19b are known to modulate both inflammation and autophagy, their upregulation may impair Nurr1’s protective role, contributing to neuroinflammatory damage in spinal cord injury. Another signaling pathway that was targeted by miR-19a and miR-19b was PI3K/AKT signaling pathway (Wang et al., **2021**) that regulates cell survival and inflammation and interacts with Nurr1 or Nur77 activity indirectly (Kurakula et al., 2014). This suggests that miR-19a and miR-19b may influence Nurr1 or Nur77 activity indirectly by targeting the PI3K/AKT signaling pathway, potentially disrupting cell survival and inflammatory regulation during spinal cord injury. Another pathway affected by miR-19a and miR-19b is the p53 signaling pathway, which controls cell death and stress responses and may also interact with Nurr1 and Nur77 during brain inflammation. Moreover, AMPK modulates inflammation, autophagy, and mitochondrial health in neural cells, processes linked to Nurr1 and Nur77 function (Zhao and Klionsky, 2011; Zhao et al., 2016). Together, these data support a model in which miR-19 family members influence transcriptional and inflammatory signaling networks relevant to microglial activation after SCI.

To assess the impact of miR-19a and miR-19b on Nurr1 and Nur77 expression following microglia activation, we measured transcript levels in microglia exposed to miR-19a or miR-19b mimics and inhibitors, with or without inflammatory stimulation (LPS and IFN-*γ*). The results showed that treatment with miR-19a and miR-19b mimics were associated with increased microglia inflammatory signaling by reducing the expression of transcription factors Nurr1 and Nur77. Several studies showed that Nurr1 has neuroprotective functions (Barneda-Zahonero et al., 2012; Pan et al., 2008; Lin et al., 2012; Zetterstrom et al., 1997; Bensinger and Tontonoz, 2009; Le et al., 2003; Amoasii et al., 2019), and also it has been shown that Nur77 has neuroprotective effects in neurodegenerative disorders (Liu et al., 2017). Both Nurr1 and Nur77 have been shown to regulate inflammatory signaling and microglial activation. These findings support their potential as therapeutic targets for neuroinflammatory and neurodegenerative disorders, including spinal cord injury.

We performed immunofluorescence staining in rat brains (uninjured and spinal cord-injured rats, as a model of neuroinflammation) to show the expression level and localization of Nurr1 and Nur77. The results showed us the expression of Nurr1 and Nur77 was decreased in the brains of SCI rats. Moreover, colocalization of Nurr1 and Nur77 with Iba-1 showed these transcription factors are expressed in microglia. A recent study has shown that Iba-1-immunoreactive microglia and Nurr1-immunoreactive cells colocalized and it confirmed our findings (Park et al., 2020). We sought to validate our *in vitro* observations using brain histological sections and a rat model of SCI. SCI is a well established model for trauma-induced brain neuroinflammation, and SCI model has been shown to cause extensive microglia activation in the brain, with increased expression of activated microglia markers (Wu, Stoica et al., 2014; Wu, Zhao et al., 2014). We found that expression of Nurr1 and Nur77 was reduced in the injured rats’ central nervous system by using a rat SCI model. These findings support the biological relevance of NR4A signaling in microglia after SCI and suggest that restoring Nurr1/Nur77 activity may represent a potential therapeutic strategy to reduce chronic neuroinflammation and neuropathic pain.

We used siRNA-mediated knockdown to suppress Nurr1 and Nur77 expression in microglia, in order to assess their regulatory roles. Silencing either transcription factor led to a marked increase in pro-inflammatory cytokine expression, indicating that reduced levels of Nurr1 and Nur77 enhance the inflammatory response in microglia.

The PPI network highlights several transcription factors, including MYC, CEBPB, RUNX1, STAT3, STAT1, KLF4, and USF1, that play critical roles in regulating neuroinflammatory pathways (Rohrer et al., 2023; Gnanaprakasam and Wang, 2017; Li et al., 2025; Cheng et al., 2018). MYC is involved in immune cell proliferation and can enhance inflammatory gene expression (Gnanaprakasam and Wang, 2017). CEBPB regulates cytokine production and is upregulated in activated glial cells during CNS injury (Li et al., 2025). RUNX1 and STAT3 are both associated with microglial activation and have been shown to influence Nurr1 and Nur77 expression, suggesting a potential indirect regulatory role of miR-19a and miR-19b on these transcription factors. STAT1, typically activated by interferons, promotes pro-inflammatory transcriptional programs in microglia. KLF4 contributes to both inflammatory signaling and neural regeneration processes, while USF1 is known to modulate genes related to immune and stress responses (Cheng et al., 2018). Together, these findings support the hypothesis that miR-19a and miR-19b may influence neuroinflammation following spinal cord injury through the modulation of key transcriptional regulators, forming a coordinated miRNA–transcription factor–nuclear receptor regulatory axis.

Despite these findings, several limitations of the current study should be acknowledged and represent important directions for future investigation. Additional mechanistic studies such as luciferase reporter assays and expanded protein-level validation would further strengthen direct targeting conclusions. In addition, STRING-based protein–protein interaction analysis is predictive and hypothesis-generating in nature. Transcription factors identified through this analysis, including MYC and STAT3, should therefore be interpreted as potential regulatory nodes requiring experimental validation by using approaches such as Co-IP, ChIP, or functional perturbation studies in future work. It is importartant to mention that although the present study focused on canonical pro-inflammatory cytokines, future studies incorporating a broader panel of M1 and M2 microglial markers, including IL-1β, iNOS, CD86, Arg1, IL-10, and CD206, will be important to more comprehensively define the effects of miR-19a and miR-19b on microglial polarization states.

From a translational perspective, targeting miR-19a and miR-19b or restoring Nurr1/Nur77 signaling may represent potential therapeutic strategies to modulate microglial activation and reduce neuroinflammation following SCI. Modulation of this regulatory axis may have clinical relevance for biomarker-guided or miRNA-based therapeutic development.

## 5 Conclusion

In summary, our findings highlight an important role of miR-19a and miR-19b in modulating neuroinflammation through the modulation of Nurr1 and Nur77, two key transcription factors involved in anti-inflammatory and neuroprotective pathways. The conserved nature of miR-19a and miR-19b and their enriched expression in immune and neural tissues suggest an evolutionarily preserved regulatory function, particularly relevant in pathological conditions such as spinal cord injury. Bioinformatic analyses further support their involvement in major signaling pathways like PI3K/AKT, FoxO, AMPK, and p53, which are crucial in inflammatory and stress responses. The observed reduction of Nurr1 and Nur77 expression in SCI models and the increased pro-inflammatory cytokine levels upon their silencing emphasize the importance of maintaining their activity in microglia to suppress neuroinflammation. Together, our results support a miR-19–transcription factor–nuclear receptor axis as a contributing mechanism in SCI-induced neuroinflammation and suggest that miR-19a and miR-19b as potential targets for future therapeutic strategies aimed at modulating neuroinflammatory process.

## Data Availability

The raw data supporting the conclusions of this article will be made available by the authors, without undue reservation.
